# Cost-effectiveness of gasless laparoscopy as a means to increase provision of minimally invasive surgery for abdominal conditions in rural North-East India

**DOI:** 10.1371/journal.pone.0271559

**Published:** 2022-08-03

**Authors:** Bryony Dawkins, Noel Aruparayil, Tim Ensor, Jesudian Gnanaraj, Julia Brown, David Jayne, Bethany Shinkins

**Affiliations:** 1 Academic Unit of Health Economics, University of Leeds, Leeds, United Kingdom; 2 Leeds Institute of Medical Research at St. James’, University of Leeds, Leeds, United Kingdom; 3 Nuffield Centre for International Health and Development, University of Leeds, Leeds, United Kingdom; 4 Karunya Institute of Technology and Science, Coimbatore, India; 5 Leeds Institute of Clinical Trials Research, University of Leeds, Leeds, United Kingdom; International School, Vietnam National University, VIET NAM

## Abstract

Laparoscopic surgery, a minimally invasive technique to treat abdominal conditions, has been shown to produce equivalent safety and efficacy with quicker return to normal function compared to open surgery. As such, it is widely accepted as a cost-effective alternative to open surgery for many abdominal conditions. However, access to laparoscopic surgery in rural North-East India is limited, in part due to limited equipment, unreliable supplies of CO_2_ gas, lack of surgical expertise and a shortage of anaesthetists. We evaluate the cost-effectiveness of gasless laparoscopy as a means to increase provision of minimally invasive surgery (MIS) for abdominal conditions in rural North-East India. A decision tree model was developed to compare costs, evaluated from a patient perspective, and health outcomes, disability adjusted life years (DALYs), associated with gasless laparoscopy, conventional laparoscopy or open abdominal surgery in rural North-East India. Results indicate that MIS (performed by conventional or gasless laparoscopy) is less costly and produces better outcomes, fewer DALYs, than open surgery. These results were consistent even when gasless laparoscopy was analysed using least favourable data from the literature. Scaling up provision of MIS through increased access to gasless laparoscopy would reduce the cost burden to patients and increase DALYs averted. Based on a sample of 12 facilities in the North-East region, if scale up was achieved so that all essential surgeries amenable to laparoscopic surgery were performed as such (using conventional or gasless laparoscopy), 64% of DALYS related to these surgeries could be averted, equating to an additional 454.8 DALYs averted in these facilities alone. The results indicate that gasless laparoscopy is likely to be a cost-effective alternative to open surgery for abdominal conditions in rural North-East India and provides a possible bridge to the adoption of full laparoscopic services.

## Introduction

Laparoscopic surgery, a minimally invasive technique to treat abdominal conditions, has been shown to produce reduced infection rates and quicker return to normal function, equivalent safety and efficacy, and possible efficiency gains for healthcare providers compared to open surgery [1, 2]. As such, laparoscopic surgery is widely accepted as a cost-effective alternative to open surgery for many patients with abdominal conditions [3–7]. However, access to laparoscopic surgery in rural parts of India is limited, in part due to limited availability of specialised equipment, including restricted supplies of CO_2_ gas, lack of surgical expertise in the technique and a shortage of anaesthetists [8, 9]. Gasless laparoscopy is a modified form of laparoscopic surgery that uses an abdominal wall lift device to create an intra-abdominal working space. There is no need for pneumoperitoneum or special laparoscopic ports, and it can be performed under spinal or general anaesthesia. A recent single-centre RCT in Delhi demonstrated non-inferiority of the technique compared to conventional laparoscopy, focusing on surgical time, intra-operative vital signs and post-operative pain [9]. However, this is the only high quality RCT to evaluate the use of gasless laparoscopy in India to date, and data from more general provision of gasless laparoscopy (i.e., outside a study setting, and relevant to more rural parts of India) is limited as the technique is not yet widespread [10]. In addition, the majority of the current evidence focuses on comparisons of conventional laparoscopy (using CO_2_ insufflation) compared to gasless laparoscopy, rather than open surgery compared to gasless laparoscopy, which is the relevant comparison in rural parts of India [1, 9, 10]. Furthermore, as gasless laparoscopy is not an aerosol generating procedure, it has been suggested as a way to facilitate provision of laparoscopic surgery when conventional laparoscopy is ruled out due to risks of potentially contaminating aerosols [11]. Consequently, gasless laparoscopy could be an alternative to conventional laparoscopy in resource limited settings, provided it can be shown to be safe and affordable compared to open surgery. Herein the term ‘minimally invasive surgery’ (MIS) is used to refer to laparoscopic surgery performed using either the conventional or gasless technique.

The aim of this study was to undertake an economic evaluation, based on current evidence to identify evidence gaps and inform future research in this area. Specific objectives were to: 1) estimate the cost-effectiveness of gasless laparoscopy compared with open surgery, as a means to increase provision of MIS; 2) undertake scenario analyses to explore the uncertainty around the effectiveness of gasless laparoscopy and the impact on the decision to provide gasless laparoscopy over open surgery in the absence of conventional laparoscopy; 3) model the impact on health outcomes and patient costs with the scale up of gasless laparoscopy.

## Methods

### Overview: Patients, interventions and outcomes

A decision tree model was developed to capture a simplified patient pathway for MIS vs open surgery for abdominal conditions amenable to laparoscopic surgery (see S2 Appendix) in rural North-east India. The model is structured so that MIS can be conventional or gasless laparoscopy. Costs and health outcomes associated with gasless laparoscopy and open surgery were compared to determine cost-effectiveness. The main outcomes were costs, evaluated from a patient perspective, and improvements in quality adjusted life, measured in terms of disability-adjusted life years (DALYs) avoided.

### Model structure

**Fig 1:**
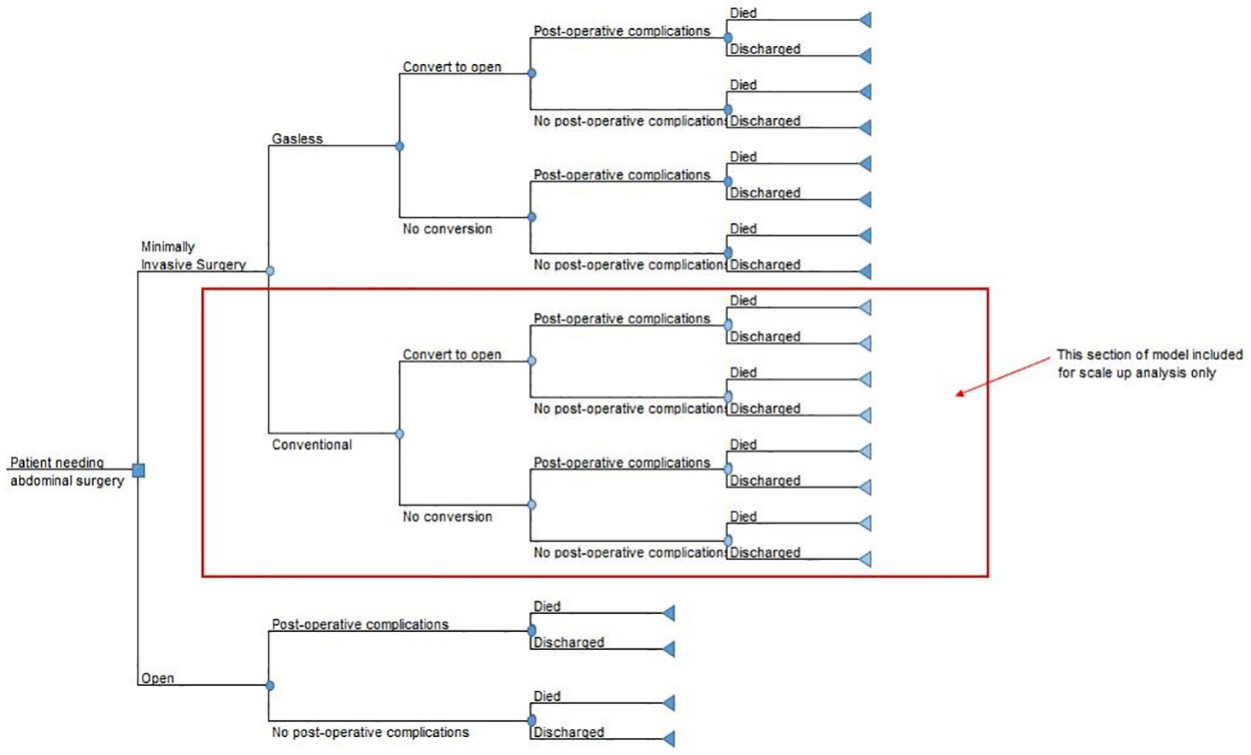
Model structure.

As most health events related to this type of surgery would occur within a relatively short time frame following surgery, and due to lack of reliable data from the rural Indian setting to facilitate long term analysis, a short time horizon limited to the hospital stay following surgery, was used for the cost-effectiveness analysis. Consequently, a decision-tree model was developed for the analysis [12]. The model structure is shown in Fig 1. Conventional laparoscopy is included within the model for the purpose of scale up analysis only. This is because in the rural Indian setting conventional laparoscopy is often not possible and as such the only realistic alternative to gasless laparoscopy is open surgery. However, a small number of conventional laparoscopies are performed in some locations where it is possible and so the option was included when modelling the cost-effectiveness of gasless laparoscopy in current practice and the impact on costs and health outcomes with scale up of gasless laparoscopy.

### Data sources for model parameters

#### Current provision of abdominal surgery

A recent survey, adapted from the WHO Emergency and Essential Surgical Care and Lancet surgery assessment [13, 14], provided data on the surgical infrastructure available and the number of different types of surgery performed in 20 healthcare facilities in North-East India. The survey focused on the availability of physical and human resources to deliver surgical care and the amount and type of surgery carried out. It included facilities in both the public and private sector and covered the districts across four states in North-East India, in which the doctors enrolled in gasless laparoscopy training and proctorship worked. Additional data on all surgeries undertaken over a 3-month period were collected directly from surgical logbooks at 12 of the facilities included in the survey. The intention had been to collect logbook information from all facilities included in the survey, however during initial survey visits this was not completed, and it was not possible to conduct follow up visits at all facilities. The facilities providing logbook data were broadly representative of the facilities in the main survey (see S1 Appendix). Using the logbook data, the number of surgeries that were amenable to MIS was identified, along with the proportion of these that were performed laparoscopically and the proportion that were performed using open surgery. This data provided an estimate of the patient population that could benefit from the scale up of gasless laparoscopy as a means to increase the availability of MIS. Data from 12 facilities where logbook data was available, were used to identify the number of MIS that were performed using the gasless technique. These data were used to inform the proportion of laparoscopic-amenable surgeries that are currently performed using gasless laparoscopy, conventional laparoscopy, and open surgery.

#### Provision of gasless laparoscopy in rural North-East India

In March 2019, the Leeds NIHR Global Health Research Group in Surgical Technologies delivered a training programme (TARGET) on laparoscopic skills and use of the gasless technique at Kolkata Medical College, India. The laparoscopic training programme involved 7 rural surgeons from Assam, Manipur, Arunachal Pradesh and Nagaland in the North-East of India. A registry was established to capture data on all abdominal operations, including gasless laparoscopy, performed in the hospitals of participating surgeons following the training programme.

Data collected in the registry up to 31/07/20 was used within the analysis to provide information relevant to the rural Indian setting on length of hospital stay, complications and conversions. At the point of data analysis, a total of 286 patients were recorded in the registry as requiring abdominal surgery, of these, 123 patients underwent gasless laparoscopy: 52 patients in Assam, 27 patients in Manipur and 44 patients in Nagaland. Details of the procedures performed using gasless laparoscopy, any complications experienced, and outcomes were recorded in the registry. Combined with data from a facility survey and the wider literature, it was used to parameterise a decision-analytic model to explore the potential cost-effectiveness of gasless laparoscopy as a means to increase provision of MIS in rural North-East India compared to current practice.

#### Clinical effectiveness

A parallel systematic review and meta-analysis was conducted comparing the clinical effectiveness of gasless vs. conventional laparoscopy or open surgery [10]. The majority of the evidence identified came from high income settings with only 4 studies identified from a LMIC setting. All of these compared gasless to conventional laparoscopy, not to open surgery. One recently published non-inferiority RCT comparing gasless to conventional laparoscopy in Delhi was identified which showed no significant differences in short-term outcomes between gasless and conventional laparoscopy [9]. Whilst this study was conducted in India, it was conducted in a well-resourced tertiary hospital in Delhi which we note is a considerably different environment to the low resource setting of rural North-East India. As such, no clinical outcome data representative of a rural Indian setting was identified.

In the base case analysis the model is therefore primarily populated with (unpublished) data from the registry of gasless laparoscopy in rural North-East India, in attempt to reflect the best available data for this setting. However, acknowledging the low grade of this evidence, and to explore the impact of using this data, alternative parameter values obtained from the systematic review were used in sensitivity analyses.

For the scale up analysis, effectiveness data for conventional laparoscopic surgery was also required to represent the small number of surgeries performed by this method in current practice. As evidence from the systematic review which came from LMICs indicated no significant difference in conversion rate, complications, or length of stay, the same values as were used for gasless laparoscopy in the primary cost-effectiveness analysis were used to parameterise the conventional laparoscopy part of the model [10].

#### Patient symptoms and outcomes following surgery

In the absence of data on patient symptoms and outcomes following surgery relevant to the rural Indian setting, expert elicitation was conducted to collect information on patients’ symptoms associated with each type of surgery, their severity and duration from surgeons working in the region. The Indian surgeons involved with the TARGET training (both as trainers and trainees) formed the pool of 9 experts that were approached in September 2020 to participate, 4 of whom provided responses. Surgeons were asked to reflect on their own experience and for each of the relevant surgeries rank the likely severity and duration of abdominal pain and motor impairment following surgery, along with the likely severity and duration of symptoms related to post-operative complications. The most commonly reported severity description for each was used to inform the relevant disability weight used within the model. Responses detailing duration of symptoms were combined to inform a plausible range for symptom durations (the minimum and maximum value elicited is reported as parameter 1 and 2, respectively, in Table 1). The mid-point of the range was used in the deterministic analysis, and uniform distributions were fitted to randomly sample from within the reported range in the probabilistic analysis. Post-operative pain recorded in a recent RCT conducted in Delhi was comparable to the severity of pain reported in the expert elicitation, however no data on severity or duration of other symptoms was available in the literature for comparison [9].

**Table 1:** Parameter values.

Parameter	Deterministic value	Distribution	Parameter 1	Parameter 2	Source
**Primary cost effectiveness analysis**					
Age	34.512	lognormal	3.491	0.316	Gasless laparoscopy registry
Probabilities—Gasless laparoscopy					
Conversion	0.114	Beta	14	123	Gasless laparoscopy registry
Post-operative complications	0.057	Beta	7	116	Gasless laparoscopy registry
Death| no conversion	0.005	Beta	746.666	149333.269	Masoomi et al, 2015
Death| conversion	0.006	Beta	178.340	29723.409	Masoomi et al, 2015
Probabilities—Open surgery					
Post-operative complications	0.237	Beta	22.041	93	Lombardo et al 2018
Death	0.017	Beta	7945.074	467357.322	Masoomi et al, 2015
Length of Hospital Stay—Gasless laparoscopy					
Length of stay, no conversion, no complications (days)	2.731	Gamma	2.681	1.018	Gasless laparoscopy registry
Additional length of stay due to conversion (days)	1.899	Gamma	9.406	0.184	Gasless laparoscopy registry
Additional length of stay due to complications (days)	2.666	Gamma	9.974	0.244	Gasless laparoscopy registry
Length of stay if die (days)	1	Fixed			Assumed
Length of Hospital Stay—Open surgery					
Length hospital stay (additional days compared with MIS)	2.84	Gamma	26.916	0.102	Aruparayil et al, 2021
Disability Weights					
Abdominopelvic problem mild	0.11	Beta	7.856	666.968	GBD 2017
Abdominopelvic problem moderate	0.114	Beta	27.320	209.366	GBD 2017
Motor impairment mild	0.01	Beta	9.011	818.517	GBD 2017
Motor impairment moderate	0.061	Beta	23.034	346.910	GBD 2017
Infectious disease mild	0.006	Beta	5.231	873.845	GBD 2017
Infectious disease moderate	0.051	Beta	21.252	395.735	GBD 2017
Duration of abdominal pain following surgery (days)					
Gasless laparoscopy	1	Uniform	1	2	Expert elicitation^1^
Converted	3	Uniform	1	5	Expert elicitation^1^
Open surgery	3	Uniform	1	5	Expert elicitation^1^
Duration of motor impairment following surgery (days)					
Gasless laparoscopy	1	Uniform	1	2	Expert elicitation^1^
Converted	3	Uniform	1	5	Expert elicitation^1^
Open surgery	3	Uniform	1	5	Expert elicitation^1^
Duration of symptoms if post op infection (days)					
Gasless laparoscopy	4	Uniform	1	7	Expert elicitation^1^
Converted	5	Uniform	3	7	Expert elicitation^1^
Open surgery	5	Uniform	3	7	Expert elicitation^1^
Patient Perspective Costs (INR)					
Gasless laparoscopy	14,000.00	Gamma	1.225	11428.571	India Facility Survey, 2019
Open laparotomy	33,031.25	Gamma	6.830	4836.298	India Facility Survey, 2019
Conversion to open	0.00	Fixed			Direct communication^2^
Medicines and supplies for surgery	5,285.71	Gamma	3.534	1495.495	India Facility Survey, 2019
Medicines and supplies for each day in hospital	2,928.57	Gamma	0.827	3540.650	India Facility Survey, 2019
Lodging per day	506.25	Gamma	3.882	130.423	India Facility Survey, 2019
Other necessities (e.g. food, laundry) not included in lodging	566.67	Gamma	1.966	288.235	India Facility Survey, 2019
Transportation per hospital stay	1,088.75	Gamma	2.366	460.172	India Facility Survey, 2019
Complications (applies to all types of abdominal surgery	0.00	Fixed			Direct communication^2^
**Additional parameters for scale up analysis**					
Probabilities—Type of surgery					
Open surgery	0.734	Beta	354	482	India Facility Survey, logbook data, 2019^4^
Minimally invasive surgery Of which:	0.266	Beta	128	482	India Facility Survey, logbook data, 2019^4^
Conventional laparoscopic surgery (given minimally invasive surgery)	0.953	Fixed			India Facility Survey, 2019^3^
Gasless laparoscopy (given minimally invasive surgery)	0.047	Fixed			India Facility Survey, 2019^3^
Probabilities—Conventional laparoscopy					
Conversion	0.114	Beta	14	123	Gasless laparoscopy registry
Post-operative complications	0.057	Beta	7	116	Gasless laparoscopy registry
Death| no conversion	0.005	Beta	746.666	149333.269	Masoomi et al, 2015
Death| conversion	0.006	Beta	178.340	29723.409	Masoomi et al, 2015
Duration of abdominal pain following surgery (days)					
Conventional laparoscopy	1	Uniform	1	2	Expert elicitation^1^
Converted	3	Uniform	1	5	Expert elicitation^1^
Duration of motor impairment following surgery (days)					
Conventional laparoscopy	1	Uniform	1	2	Expert elicitation^1^
Converted	3	Uniform	1	5	Expert elicitation^1^
Duration of symptoms if post op infection (days)					
Conventional laparoscopy	4	Uniform	1	7	Expert elicitation^1^
Converted	5	Uniform	3	7	Expert elicitation^1^
Patient Perspective Costs (INR)					
Conventional laparoscopy	25857.14286	Gamma	8.327656329	3104.972376	India Facility Survey, 2019

^1^Parameters 1 and 2 are minimum and maximum values reported, respectively

^2^From a patient cost perspective there are no additional costs associated with complications as in this context in the event of unexpected complications additional costs are absorbed by the hospital

^3^Facility survey of 20 healthcare facilities across 4 states in North-East India

^4^Logbook data on all surgeries over 3 months was obtained from 12 facilities who took part in the main facility survey

Where possible, data on patient outcomes from the gasless laparoscopy registry were used. This included conversion rates, complication rates and length of hospital stay. As part of the expert elicitation to obtain information about patient outcomes and symptoms following surgery, surgeons were asked about complications patients might experience. Their responses indicated that the most common complication was infection and while post-operative bleeding and complications due to perforation were plausible, they occurred very infrequently and so were unable to provide detail on likely patient outcomes in this case. This was supported by data from the registry in which the only post-operative complications reported were infection or post-operative pain. Consequently, for the purposes of the decision model, complications were assumed to be infections. Mortality rates used within the model were based on an estimate from the literature [15], as there was insufficient data from the gasless laparoscopy registry due to only one death recorded.

Where data were lacking from the sources described above, data to inform parameters was sought from the wider literature. All model parameters are presented in Table 1.

### Outcomes

The primary outcome for the cost-effectiveness analysis was disability adjusted life years (DALYs). DALYs were calculated as the sum of years of life lost (YLL) to premature death and the years lived with disability (YLD):

DALY=YLL+YLD

YLL is calculated as:

YLL=N*L

Where *N* is the number of deaths, and *L* is the remaining life expectancy, in years, at the age of death.

YLD is calculated as:

YLD=(I*D)*W

Where *I* is the number of incident cases of a particular condition, *D* is the duration of disability from a particular condition, and W is the disability weight associated with the condition.

DALYs were calculated using the life expectancy standard loss function, the relevant disability weights from the Global Burden of Disease Study 2017 which were applied based on the expected patient symptoms and outcomes for each patient pathway (e.g. with/ without complications) [16], and information on duration of patient symptoms following surgery obtained by expert elicitation (as described above). Relevant disability weights and parameters for duration of symptoms used to calculate DALYs are presented in Table 1. YLLs due to premature death were discounted at a rate of 3%. As the duration of disability following surgery was less than 1 year, no discounting was used in YLD calculation. No age-weighting was used in the calculation of DALYs.

### Costs

Cost estimates were based on data from a recent healthcare facility survey conducted in North-East India. All costs used in the analysis are presented in Table 1. Costs were measured in Indian Rupees (INR) and converted to US dollars (USD) using exchange rate 1 USD = 76.03 INR for comparison with the cost-effectiveness threshold.

### Cost-effectiveness threshold

Historically, several thresholds have been proposed to guide cost-effectiveness analysis. For example, until 2016, WHO recommended a cost-effectiveness threshold of 1–3 times GDP per capita (although this is no longer recommended per se) [17, 18], while an empirical estimate of the threshold based on the opportunity cost of resource allocation decisions in India was estimated in 2015 as 264–363 USD per DALY averted, equivalent to 17–23% GDP per capita [19].

We therefore present cost-effectiveness results against a range of thresholds on the cost-effectiveness acceptability curve (CEAC). As an aid to decision-making, we also mark on the cost-effectiveness plane thresholds representing 17%, 23%, 100% and 300% GDP per capita, representing the thresholds proposed above. However, decision makers should compare the cost-effectiveness results to the threshold that is relevant to their decision-making context.

### Cost-effectiveness analysis

The primary analysis explored the cost-effectiveness of gasless laparoscopy compared with open surgery for abdominal surgeries amenable to laparoscopic surgery in rural North-East India. The analysis was undertaken from a patient perspective as, in this setting, healthcare is paid for almost exclusively by patients. This included direct costs incurred by patients associated with the hospital stay as well as indirect costs such as food and transport for the patient and family [20]. Analysis from a healthcare provider perspective was also considered, but there was insufficient reliable data to facilitate this.

Mean costs and DALYs were calculated for each of the surgical options and incremental cost-effectiveness ratios (ICER) were estimated as:

$$ICER=\frac{Incremental\;cost}{Incremental\;DALYs\;averted}$$

Where, incremental cost is the expected cost associated of undergoing gasless laparoscopy minus the expected cost of undergoing open surgery; and incremental DALYs averted is the expected DALYs if undergoing open surgery minus the expected DALYs if undergoing gasless laparoscopy.

The ICER was compared with the cost-effectiveness threshold(s) to determine cost-effectiveness [20, 21].

### Probabilistic sensitivity analysis

Probabilistic sensitivity analysis was undertaken using Monte-Carlo simulations to evaluate the uncertainty within the model. Results were plotted on the cost-effectiveness plane, showing the uncertainty around the point estimate of cost-effectiveness, and the cost-effectiveness acceptability curve (CEAC), showing the probability of cost-effectiveness over a range of cost-effectiveness threshold values [21].

### Sensitivity analyses

A series of sensitivity analyses were undertaken to explore the impact of assumptions made in the model. A key concern in the development of the model was the lack of published data which was representative of the rural India setting. As such unpublished data from a registry of gasless laparoscopies in the region was used in the base case analysis. The impact of using data from the registry to parameterise the model was explored in sensitivity analysis which instead used data from the published literature. Best- and worst-case scenarios were constructed based on available data on conversion rates, complications and length of stay from the literature, informed by a parallel systematic review [10]. The best-case scenario used the values for each of these parameters that were most favourable to gasless laparoscopy i.e., lowest reported values. Conversely, the worst-case scenario used the values least favourable to gasless laparoscopy e.g., highest reported values.

Sensitivity analyses were also undertaken to explore the uncertainty around the cost of gasless laparoscopy using highest and lowest values for cost of gasless laparoscopy as reported in the India facility survey, 2019. In addition, sensitivity analyses were undertaken to explore uncertainty around the DALYs accrued by patients using extreme values (high and low values) for disability weights informed by the reported confidence intervals around disability weights from the Global Burden of Disease Study, 2017. For each, the analysis was re-run using relevant parameters to identify the impact on results. Parameters used in the sensitivity analyses are presented in Table 2 (base case values were used for any parameters not listed).

**Table 2:** Parameter values—Sensitivity analyses.

Parameter	Value	Source
***Gasless laparoscopy—Best-case scenario***		
*Gasless laparoscopy parameters*		
Probability conversion	0	[22–25]
Probability complications	0	[26–33]
Length of hospital stay (days)	1	[32–34]
***Gasless laparoscopy—Worst-case scenario***		
*Gasless laparoscopy parameters*		
Probability conversion	0.181818	[30, 35]
Probability complications	0.4	[20]
Length of hospital stay (days)	8	[30]
***Lower cost for gasless laparoscopy***		
Cost of gasless laparoscopy (INR)	2,000	Lowest reported value, India Facility Survey, 2019
***Higher cost for gasless laparoscopy***		
Cost of gasless laparoscopy (INR)	30,000	Highest reported value, India Facility Survey, 2019
***Lower disability weights***		
Abdominopelvic problem mild	0.005	[16]
Abdominopelvic problem moderate	0.078	[16]
Motor impairment mild	0.005	[16]
Motor impairment moderate	0.04	[16]
Infectious disease mild	0.002	[16]
Infectious disease moderate	0.034	[16]
***Higher disability weights***		
Abdominopelvic problem mild	0.021	[16]
Abdominopelvic problem moderate	0.159	[16]
Motor impairment mild	0.019	[16]
Motor impairment moderate	0.089	[16]
Infectious disease mild	0.012	[16]
Infectious disease moderate	0.074	[16]

### Analysis of impact of scale-up on health outcomes

Scale up analysis was undertaken to model the impact on costs and health outcomes as the provision of gasless laparoscopy increases. The proportion of laparoscopic-amenable surgeries currently performed using gasless laparoscopy, conventional laparoscopy and open surgery were incorporated into the model, demonstrating the cost-effectiveness of MIS in current practice. The proportion of laparoscopic-amenable surgeries performed by each method were then varied within the model to reflect increasing provision of gasless laparoscopy, i.e., increasing the number of laparoscopic amenable surgeries performed as gasless laparoscopy and reducing the number performed as open surgery. Starting with 100% of surgeries set to be performed by open surgery and 0% by MIS, the proportions were then adjusted at intervals of 10% and the impact on patient costs and outcomes was recorded. The availability/capacity to provide conventional laparoscopic surgery was assumed to remain constant at 2019 levels and a fixed capacity parameter for conventional laparoscopic surgery was used to reflect this. Where the number of surgeries performed as MIS was set to a value below this capacity parameter, all MIS surgeries were modelled as being performed by conventional laparoscopy. Where the number of surgeries performed as MIS was set to a value above the capacity parameter the additional MIS surgeries were modelled as being performed using gasless laparoscopy—thus representing the scale up of gasless laparoscopy to increase provision of MIS. This assumes that all surgeries amenable to be performed laparoscopically would be amenable to be performed using the gasless technique. Whilst gasless surgery would not be used for complex surgeries, in this context those operations would not currently be performed and so in the North-East Indian setting operations amenable to laparoscopic surgery could also be performed using the gasless technique.

### Ethics

Ethical approval for the registry of gasless laparoscopies was obtained from the School of Medicine Research Ethics Committee at the University of Leeds, UK (reference MREC 18–100) and the University Research Ethics Committee at the Martin Luther Christian University, India (reference VI/I(8)/UREC/EA/272/2015-6111). Informed written consent was obtained from patients to be included in the registry. Ethical approval to explore the provision of surgical care for rural patients in North-East India was obtained from the School of Medicine Research Ethics Committee at the University of Leeds, UK (reference MREC 17–078) and Sigma-IRB, India (reference 10077/IRB/D/18-19). Informed written consent was obtained from the board of each hospital to participate in the study.

## Results

### Cost-effectiveness analysis

The primary cost-effectiveness results are presented in Table 3. Gasless laparoscopy is less costly and produces better outcomes, less DALYs, compared to open surgery. Gasless laparoscopy therefore dominates open surgery as the more cost-effective surgical option. As gasless laparoscopy dominates open surgery in the base case deterministic analysis, comparison with a cost-effectiveness threshold is not necessary.

**Table 3:** Cost-effectiveness results.

Surgery	Expected cost (USD)	Incremental cost (USD)	Expected DALYs	Incremental DALYs averted	ICER: cost per DALY averted
**Base case analysis**					
Open surgery	817.09		0.456		Gasless laparoscopy dominates
Gasless laparoscopy	413.54	-403.55	0.135	0.321	
**Sensitivity analyses**					
*GILLS best-case scenario*					
Open surgery	817.09		0.456		Gasless laparoscopy dominates
Gasless laparoscopy	313.16	-503.93	0.134	0.322	
*GILLS worst-case scenario*					
Open surgery	1039.14		0.456		Gasless laparoscopy dominates
Gasless laparoscopy	698.02	-341.12	0.139	0.317	
*Lower cost for GILLS*					
Open surgery	817.09		0.456		Gasless laparoscopy dominates
Gasless laparoscopy	255.7	-561.38	0.135	0.321	
*Higher cost for GILLS*					
Open surgery	817.09		0.456		Gasless laparoscopy dominates
Gasless laparoscopy	623.98	-403.55	0.135	0.321	
*Lower disability weights*					
Open surgery	817.09		0.456		Gasless laparoscopy dominates
Gasless laparoscopy	413.54	-403.55	0.135	0.321	
*Higher disability weights*					
Open surgery	817.09		0.457		Gasless laparoscopy dominates
Gasless laparoscopy	413.54	-403.55	0.135	0.322	
**Cost-effectiveness of minimally invasive surgery vs open surgery in current practice**					
Open surgery	817.09		0.456		Minimally invasive surgery dominates
Minimally invasive surgery	562.18	-254.91	0.135	0.321	

**Fig 2:**
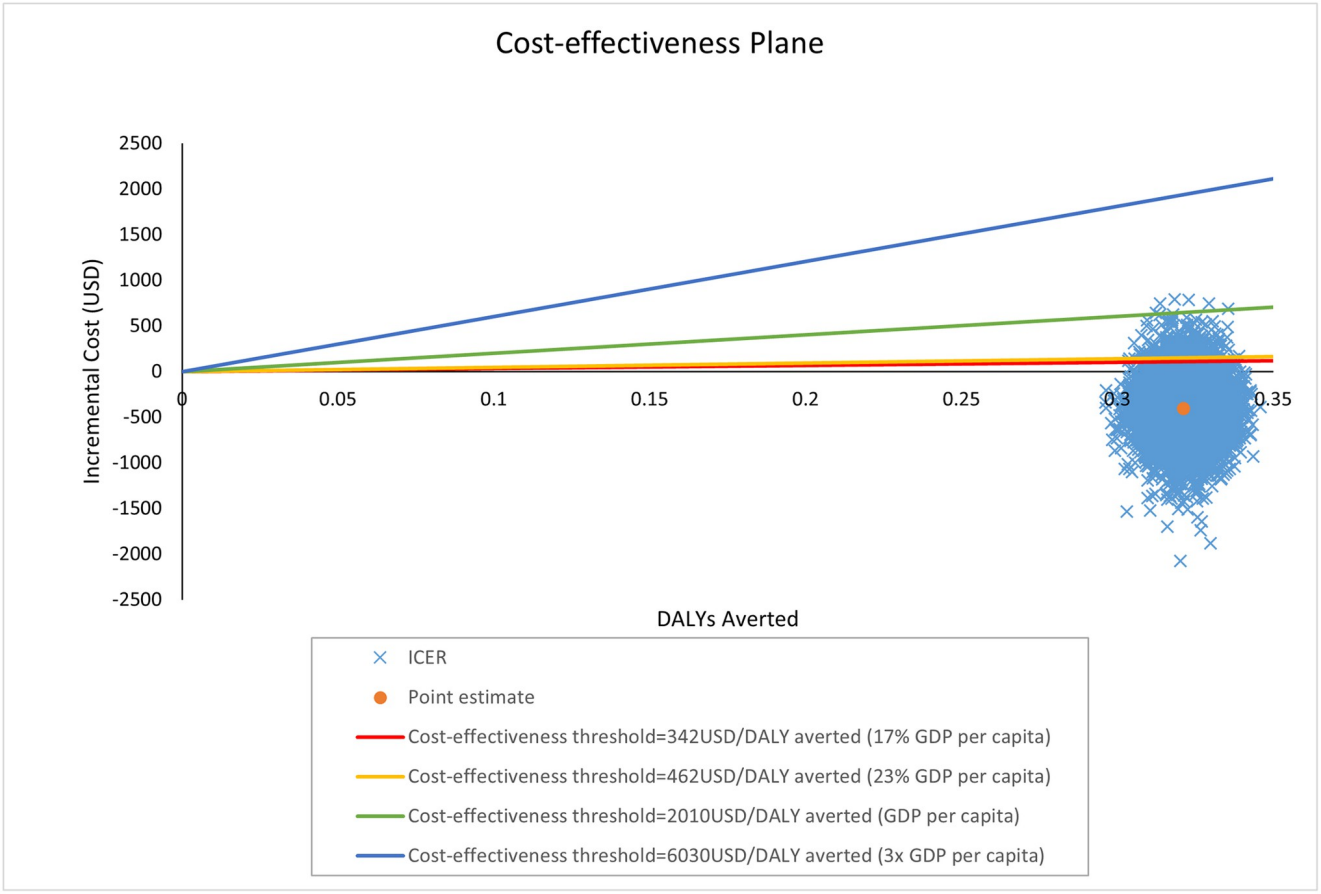
Cost-effectiveness plane.

**Fig 3:**
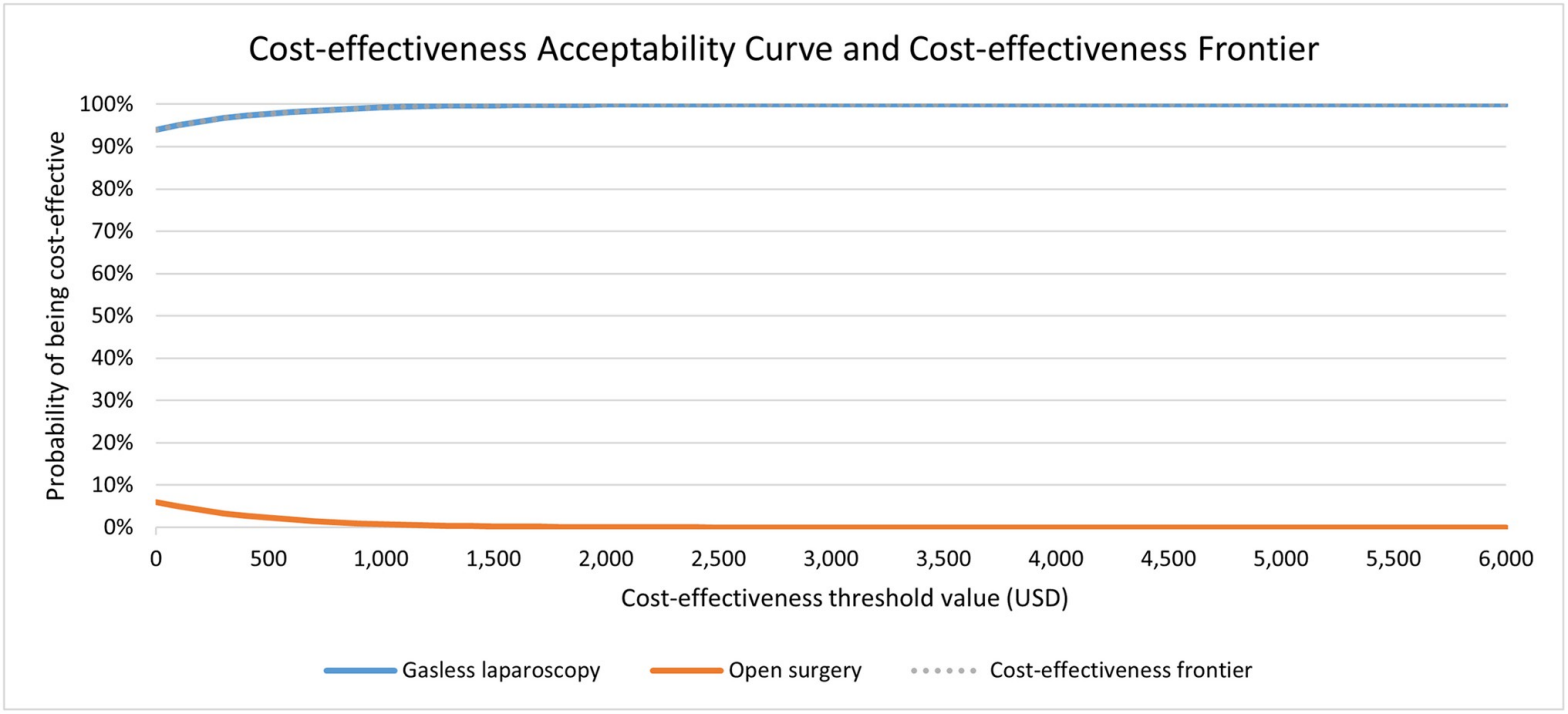
Cost-effectiveness acceptability curve.

Results of the probabilistic sensitivity analysis are presented on the cost-effectiveness plane in Fig 2. The cloud of point estimates lies entirely to the right of the y-axis indicating some degree of certainty that gasless laparoscopy produces better outcomes for patients than open surgery. As the cloud of point estimates straddles the x-axis, this illustrates the uncertainty around the costs associated with gasless laparoscopy vs open surgery. At a cost-effectiveness thresholds of 342 and 462 USD per DALY averted (representing opportunity cost [19]) the majority of points (97% and 98%, respectively) lie below the line indicating a 97–98% probability gasless laparoscopy is the most cost-effective surgery option. This increases to 100% at thresholds representing 1- and 3-times GDP per capita of 2010 USD/ DALY averted and 6030 USD/DALY averted, respectively. The probability that gasless laparoscopy is the more cost-effective option is presented over a range of cost-effectiveness threshold on the CEAC in Fig 3.

### Sensitivity analyses

The results of the sensitivity analyses are presented in Table 3. The results of the main analysis were robust to all sensitivity analyses explored. This indicates that gasless laparoscopy produces better outcomes at lower costs to patients than open surgery. This conclusion was robust even in the worst-case scenario which used the least favourable data on conversion rate, complications and length of stay associated with gasless laparoscopy as identified by a recent systematic review of the literature [10].

### Scale up analysis

**Fig 4:**
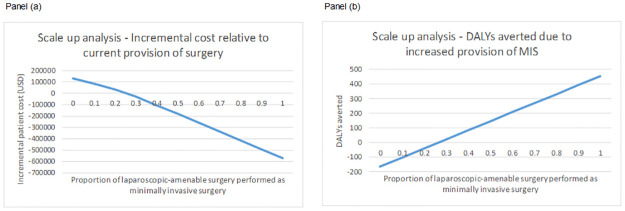
Scale up analysis.

The cost-effectiveness of MIS compared with open surgery in current practice is presented in Table 3 and results of the scale up analysis are presented in Fig 4. Scale up of the provision of MIS through increased provision of gasless laparoscopy would reduce the cost burden to patients (Panel a) and increase the number of DALYs averted (Panel b). Based on a sample of 12 facilities in rural North-East India, if scale up of the provision of gasless laparoscopy was achieved to a level where all surgeries that were amenable to laparoscopic surgery were performed as such (rather than the high proportion currently performed as open surgery), 64% of DALYS related to surgery in this patient group could be averted. Based on the surgeries performed in these facilities in 2019, this would equate to an additional 454.8 DALYs averted in these facilities alone.

## Discussion

There is limited access to laparoscopy in rural regions of North-East India; open surgery is still carried out for the majority of abdominal surgeries potentially amenable to laparoscopy. Our analysis suggests that increased provision of gasless laparoscopy and thus access to MIS would be more cost-effective than current surgical practice. This conclusion held up even when data least favourable to gasless laparoscopy identified from the published literature was used within the analysis, indicating that gasless laparoscopy could serve as a viable bridge to full laparoscopic services by improving patient outcomes and quality of life, and reducing costs compared to open surgery. Furthermore, as a high proportion of the poorest (lowest socio-economic groups) live in rural regions of North-East India [36], reducing the cost burden to patients whilst improving their health outcomes has potential implications for reducing inequalities. Formal evaluation of the impact of increased access to minimally invasive surgery on inequalities was not possible within this study due to a lack of relevant data. However, this should be considered as an important topic for future research.

A key strength of this modelling exercise is the use of real-world, region-specific data to inform model parameters. We had access to the results of a recent survey of 20 healthcare facilities in North-East India who each provided data on the surgical infrastructure available and the number of different types of surgery performed. Following a recent surgical training programme in rural North-East India, our wider global health research team have set-up a live registry of gasless laparoscopies, recording key clinical outcomes such as conversion rate and complications, length of hospital stay and the cost of the surgery to the patient.

The short time-horizon is a limitation of this analysis. Limiting to the hospital stay means any complications post-discharge, which could be around 30% of all complications [37], are not captured within this analysis. However, this was necessary in the absence of relevant data to inform longer-term analysis as all Indian evidence currently available comes from studies with only short-term follow up. Furthermore, the current evidence that does exist indicates there is no difference in overall complications between gasless laparoscopy and open surgery [10]. There was also limited data available on symptom duration and we therefore had to elicit expert advice from a limited number of surgeons in this setting on the likely average duration of symptoms. Experts were asked about the duration of each symptom following surgery in days and hours, however all responded with whole days rather than hours which may be a simplification. This data was used to inform the DALY calculations. We explored the robustness of the results to the DALY estimates included in the model and the results were consistent regardless of the changes. However, further data will continue to be gathered via the registry to further refine these estimates. In addition, there was no available evidence on conversion and complication rates for conventional laparoscopy in a rural North-East India setting. The assumption that conventional and gasless laparoscopy are equivalent (based on [9]) may have led to an overall underestimate of the DALYs averted in current practice if conventional laparoscopy in fact results in better outcomes than gasless laparoscopy.

Our results indicate that improving access to MIS in the rural North-East India region is essential to improve the affordability of abdominal surgery and, ultimately, reduce the inequalities in outcomes between rural and urban settings. Gasless laparoscopy offers a viable approach to achieving this whilst access to conventional laparoscopy is limited.

## Supporting information

S1 Appendix Comparison of facilities included in facility survey and subset providing logbook data. (DOCX)

S2 Appendix Abdominal surgeries amenable to laparoscopic surgery in rural North-East India. (DOCX)
