# Supplementary material for: Cost-effectiveness of gasless laparoscopy as a means to increase provision of minimally invasive surgery for abdominal conditions in rural North-East India
Source: PLoS One. 2022 Aug 3;17(8):e0271559. doi: 10.1371/journal.pone.0271559 (PMC9348710; doi:10.1371/journal.pone.0271559)
Supplement: S1 Appendix — (DOCX) [file pone.0271559.s001.docx]

**S1 Appendix – Comparison of facilities included in facility survey and subset providing logbook data**

Table 1 – Comparison of districts

| District | Frequency of facilities in survey | Frequency of facilities in logbook data |
| --- | --- | --- |
| Bongaigaon | 1 (5%) | - |
| Chirang | 2 (10%) | 2 (16.7%) |
| Churachandpur | 2 (10%) | 2 (16.7%) |
| Dimapur | 12 (60%) | 8 (66.7%) |
| Kohima | 1 (5%) | - |
| West Kameng | 2 (10%) | - |

Table 2 – Comparison of states

| State | Frequency of facilities in survey | Frequency of facilities in logbook data |
| --- | --- | --- |
| Arunachal Pradesh | 2 (10%) | - |
| Assam | 3 (15%) | 2 (16.7%) |
| Manipur | 2 (10%) | 2 (16.7%) |
| Nagaland | 13 (65%) | 8 (66.7%) |

Table 3 – Comparison of ownership

| Ownership | Frequency of facilities in survey | Frequency of facilities in logbook data |
| --- | --- | --- |
| Government | 5 (25%) | 4 (33.3%) |
| Mission | 1 (5%) | 1 (8.3%) |
| Private | 14 (70%) | 7 (58.3%) |

Table 4 – Comparison of level of facility

| Level of facility | Frequency of facilities in survey | Frequency of facilities in logbook data |
| --- | --- | --- |
| Community health centre | 1 (5%) | 1 (8.3%) |
| District hospital | 4 (20%) | 3 (25%) |
| Sub-district hospital | 2 (10% | 1 (8.3%) |
| Teaching hospital | 1 (5%) | 1 (8.3%) |
| Tertiary hospital | 6 (30%) | 3 (25%) |
| Other | 6 (30%) | 3 (25%) |

Table 5 - Comparison of facility characteristics

| Characteristic | Facilities in survey | | Facilities in logbook data | |
| --- | --- | --- | --- | --- |
|  | Mean (SD) | Min, Max | Mean (SD) | Min, Max |
| Admissions | 4386.95 (6103.71) | 300, 27000 | 4994.92 (7603.98) | 600, 27000 |
| Inpatient beds | 55.75 (41.36) | 11, 150 | 71.08 (46.83) | 15, 150 |
| Surgical beds | 32.4 (38.83) | 2, 120 | 46.5 (43.69) | 2, 120 |
| Population served | 378493.8 (535182.6) | 0, 1700000 | 462906.4 (557227.4) | 44877, 1700000 |
