# Supplementary material for: Cost-effectiveness of gasless laparoscopy as a means to increase provision of minimally invasive surgery for abdominal conditions in rural North-East India
Source: PLoS One. 2022 Aug 3;17(8):e0271559. doi: 10.1371/journal.pone.0271559 (PMC9348710; doi:10.1371/journal.pone.0271559)
Supplement: S2 Appendix — (DOCX) [file pone.0271559.s002.docx]

**S2 Appendix – Abdominal surgeries amenable to laparoscopic surgery in rural North-East India**

Appendicectomy

Cholecystectomy

Diagnostic Exploration

Gastrostomy

Hernia repair

Hysterectomy

Jejunostomy feeding tube

Laparotomy

Salpingo-oophorectomy

Myomectomy

Ovarian Cystectomy

Ovariotomy

Repair of perforations: for example, perforated peptic ulcer, typhoid ileal perforation

salpingectomy

Tubal ligation

Vaginal hysterectomy
